# Nicotinamide mononucleotide supplementation enhances aerobic capacity in amateur runners: a randomized, double-blind study

**DOI:** 10.1186/s12970-021-00442-4

**Published:** 2021-07-08

**Authors:** Bagen Liao, Yunlong Zhao, Dan Wang, Xiaowen Zhang, Xuanming Hao, Min Hu

**Affiliations:** 1 Department of Sports Medicine, Guangzhou Sport University, Guangzhou, 510150 China; 2 Guangdong Physical Fitness and Health Management Association, Guangzhou, 510310 China; 3 Guangzhou Institute of Sports Science, Guangzhou, 510620 China; 4 South China Normal University, Guangzhou, 510631 China

**Keywords:** Exercise training, NMN supplementation, Ventilatory threshold, Aerobic capacity

## Abstract

**Background:**

Recent studies in rodents indicate that a combination of exercise training and supplementation with nicotinamide adenine dinucleotide (NAD^+^) precursors has synergistic effects. However, there are currently no human clinical trials analyzing this.

**Objective:**

This study investigates the effects of a combination of exercise training and supplementation with nicotinamide mononucleotide (NMN), the immediate precursor of NAD^+^, on cardiovascular fitness in healthy amateur runners.

**Methods:**

A six-week randomized, double-blind, placebo-controlled, four-arm clinical trial including 48 young and middle-aged recreationally trained runners of the Guangzhou Pearl River running team was conducted. The participants were randomized into four groups: the low dosage group (300 mg/day NMN), the medium dosage group (600 mg/day NMN), the high dosage group (1200 mg/day NMN), and the control group (placebo). Each group consisted of ten male participants and two female participants. Each training session was 40–60 min, and the runners trained 5–6 times each week. Cardiopulmonary exercise testing was performed at baseline and after the intervention, at 6 weeks, to assess the aerobic capacity of the runners.

**Results:**

Analysis of covariance of the change from baseline over the 6 week treatment showed that the oxygen uptake (VO_2_), percentages of maximum oxygen uptake (VO_2max)_, power at first ventilatory threshold, and power at second ventilatory threshold increased to a higher degree in the medium and high dosage groups compared with the control group. However, there was no difference in VO_2max_, O_2_-pulse, VO_2_ related to work rate, and peak power after the 6 week treatment from baseline in any of these groups.

**Conclusion:**

NMN increases the aerobic capacity of humans during exercise training, and the improvement is likely the result of enhanced O_2_ utilization of the skeletal muscle.

**Trial registration number:**

ChiCTR2000035138.

**Supplementary Information:**

The online version contains supplementary material available at 10.1186/s12970-021-00442-4.

## Introduction

Nicotinamide adenine dinucleotide (NAD^+^) is pivotal to physiological processes, not only as the coenzyme of cellular oxidation–reduction reactions but also for the activation of NAD^+^-consuming enzymes, such as sirtuins, poly-ADP-ribose polymerases (PARPs), cADP-ribose synthases, NADase (CD38), and mono-ADP-ribose transferases (ARTs) [1]. NAD^+^ participates in more than 50% of all physiological processes, including mitochondrial biogenesis, cardiovascular protection, neuroprotection, oxidative stress, DNA damage repair, stem cell rejuvenation, and inflammation [2]. Low NAD^+^ levels are associated with age-associated physical disability and diseases, such as metabolic syndrome and cardiovascular disease [1–3]. The NAD^+^ salvage pathway, which utilizes NAD^+^ precursors, is the predominant mechanism for maintaining cellular NAD^+^ levels in rodents and humans [1]. Among a diverse range of NAD^+^ precursors, nicotinamide riboside (NR) and nicotinamide mononucleotide (NMN), two forms of water-soluble vitamin B_3_, are the two that have been studied in a surge of NAD^+^ precursor research in recent years due to their safety. Supplementation with NR and supplementation with NMN have shown a dose-dependent increase in cellular NAD^+^ levels in a variety of tissues in rodents and humans [4–6]. Accordingly, replenishment of depleted NAD^+^ pools by NR (mainly at the dose of 300 or 400 mg/kg/d in mice or rats) or NMN (mainly at doses of 100–500 mg/kg/d in mice) alleviates pathologic states in rodent models of age- or diet-induced decline in physical function, metabolic dysfunctions, neurodegenerative diseases, cardiomyopathy, and myocardial and cerebral ischemic injury [2, 6]. In addition, NMN supplementation improves energy expenditure, physical activity, and neuronal function in a dose-dependent manner [7, 8]. Nevertheless, clinical studies have shown that the effects of NR (dosages from 500 mg/d to 2000 mg/d; durations of 1 day to 3 months) on skeletal muscle, cardiovascular function, and physical function in the elderly and obese are limited [4]. However, the NAD^+^ precursor nicotinic acid (NA), an effective pharmacological drug with indications for lowering triacylglycerol levels and dilating blood vessels [9], significantly improved skeletal muscle function in healthy individuals [10] and in patients with adult-onset mitochondrial myopathy [11, 12]. NR and NMN have many similar pharmacological effects, but they are also different in a number of aspects. For example, in contrast to NMN, NR was observed to be unstable in rodent plasma [13]. Furthermore, NMN improved cardiac function in patients with Friedreich’s ataxia (FRDA) cardiomyopathy, whereas NR did not [14].

Regular exercise improves aerobic capacity, cardiovascular health, metabolic health, and physical function [15, 16], but many of such improvements may be NAD^+^/sirtuins-related [17, 18]. Exercise training increases the expression of nicotinamide phosphoribosyltransferase (NAMPT), the rate-limiting enzyme in the NAD^+^ salvage pathway, as well as the levels of NAD^+^ and activity of sirtuins [19, 20]. Endurance athletes show a high NAMPT expression in skeletal muscle [21]. One study that used a mouse model of maternal obesity [22] reported that 18-day NMN injection and 9 weeks of exercise had similar benefits for reversing metabolic dysfunction and showed that NMN had stronger effects on hepatic fat metabolism than did exercise. Deletion of PARP-1 or CD38, or the inhibitor of CD38 or PARP-1, improves mitochondrial function and endurance performance in mice [23–25]. Furthermore, overexpression of muscle NAMPT in combination with exercise in wild-type mice augmented exercise endurance, maximum oxygen uptake (VO_2_max), and mitochondrial respiratory capacity compared with exercise training alone [23, 26]. NR (400 mg/kg/d) and NMN (500 mg/kg/d) with exercise training increased endurance performance in healthy young [27] and elderly [17] mice. Nevertheless, a decrease in endurance performance from NR (300 mg/kg/d) in combination with swimming training was shown in young rats [28]. At present, the effect of the combination of exercise and NMN supplementation on cardiovascular fitness in healthy humans has not been reported. Cardiopulmonary exercise test (CPET) is the gold-standard method for assessing aerobic fitness. In this study, we conducted a six-week randomized, double-blind, four-arm, placebo-controlled clinical trial to investigate the effect of different doses of NMN supplementation on cardiovascular fitness.

## Methods

### Subjects

This study was performed at the Key Laboratory of Exercise and Health Promotion of Guangzhou Sport University and was approved by the ethics committee of Guangzhou Sport University (ethics approval number 2020 LCLL-003). Forty-eight healthy recreationally trained runners (40 males and 8 females, aged 27–50 years, with regular exercise years of 1–5 years) from the Guangzhou Pearl River running team were recruited for the study. All participants were healthy and nonsmokers who did not drink caffeine or alcoholic drinks and had no prior use of medication or supplemental nutrients. Furthermore, all participants gave written informed consent to be included in the study before the initiation of the study.

### Study design

The study was a double-blind, randomized controlled trial, and it is registered in the Chinese Clinical Trial Registry (ChiCTR2000035138 at http://www.chictr.org.cn/). Participants were randomly assigned to one of four groups (each group included ten male participants and two female participants). Randomization was stratified for gender. Allocation to nutritional supplementation with NMN (treatment groups) or placebo (control group) was concealed to the participants, support staff, and investigators, except for the quality specialists, during the course of the study.

### Supplementation

All of the participants were instructed to not change their habitual diet and daily living routines, and to refrain from caffeine during the study. The oral supplementation of powder (with or without NMN) lasted for 6 weeks. The lower dosage group received one bag with 150 mg NMN powder twice daily (Bid), the medium dosage group received one bag with 300 mg NMN powder Bid, the high dosage group received one bag with 600 mg NMN powder Bid, and the control group received one bag with matching placebo powder containing no NMN Bid. The placebo powder was composed of cranberry powder and maltodextrin, and all bags with different NMN were identical in weight, size, shape, and color. All the materials, including the placebo powder, were provided by GeneHarbor (Hong Kong) Biotechnologies Ltd. Participants were asked to take their supplement (one bag at breakfast and one at lunch or in the afternoon) before training. To record the consumption count, any remaining bags were returned to the study supervisor to ensure compliance to the supplementation protocol every weekend and at the end of the study.

### Training

All participants actively trained during the study period by adhering to an exercise program. The exercise program consisted of 6 weeks of aerobic exercise (running and cycling), with a single exercise session lasting 40–60 min. The exercise program called for 5–6 of these sessions per week. Furthermore, all participants were required to run 3–4 times per week and ride the bicycle twice per week. The exercise intensity was monitored by heart rate (HR) measurements based on the results of the CPET at baseline. The target HR ranges of exercise corresponded to 80–100% VO_2_ (80–90% VO_2_ for cycling, 90–100% VO_2_ for running) at first ventilatory threshold (VT_1_) during the first 2 weeks, 90–110% VO_2_ (90–100% VO_2_ for cycling, 100–110% VO_2_ for running) at VT_1_ during the middle 2 weeks, and 90–120% VO_2_ (90–110% VO_2_ for cycling, 100–120% VO_2_ for running) at VT_1_ in the last 2 weeks. The training sessions were conducted in the afternoon or in the early evening on working days and in the morning on weekends. The target HR range was monitored by sports watch (Garmin Forerunner 245) during exercise and study personnel supervised the training program throughout the study.

### Measurement of results

All participants were evaluated by CPET at baseline and at the end of the six-week experimental intervention period. On testing day, participants came to the laboratory, which had a room temperature set between 20 °C and 25 °C. Testing began at least 2.5 h after a normal meal, and participants could not have caffeine or alcohol for at least 12 h before the measurements.

### Anthropometric data

Height and body mass were assessed by a height-weight meter. Body composition (body fat %) and free-fat mass (FFM) were assessed by bioelectrical impedance analysis (Seca mBCA-115, Germany). Body mass index (BMI) was calculated as weight(kg)/Height(m)^2^.

### Cardiopulmonary endurance performance

Cardiopulmonary endurance performance was evaluated by CPET. The main measuring parameters include cardiovascular parameters (VO_2_, O_2_-pulse, VO_2_-related to work rate), ventilatory parameter (V_E_), metabolic parameters (respiratory exchange ratio, VT_1_, and VT_2_) and exercise capacity parameters (workload and power). For the determination of above parameters, the participants were required to complete an incremental ramp exercise test until exhaustion was reached, as indicated on a cycloergometer (Ergoline ErgoSelect 200, Germany). For males, the starting workload was 50–100 W, and there was a continuous increase of 20–30 W per minute. For females, the starting workload was 50–75 W, and there was a continuous increase of 15–25 W per minute. The test was terminated when any three of the five following criteria were met: volitional fatigue, as indicated by an inability to maintain a set rate after verbal encouragement was given; HR failing to increase with the increasing workload; an increase in VO_2_ < 150 mL/min despite the workload increasing; a respiratory exchange ratio (RER) ≥1.10; a Borg rating of perceived exertion > 17. The gas analyzer system was calibrated according to the manufacturer’s recommendations before each test. In this test, a cardiorespiratory function test system (CORTEX MetaLyzer®3B, Germany) recorded the HR, heart rate reserve (HRR), RER, workload, power, oxygen consumption (VO_2_), carbon dioxide production (VCO_2_), expired minute volume (V_E_), partial pressure of end-tidal O_2_ (PETO_2_), and partial pressure of end-tidal CO_2_ (PETCO_2_). A 12-lead electrocardiogram (ECG, Cardio 300 UBE00880, Germany) was continuously recording during the test. Blood pressure, including systolic blood pressure and diastolic blood pressure, was measured every 3 min during the cycling.

### Data collection and reduction

Measurements of the parameters [VO_2max_, V_Emax_, HR_max_, HRR, peak workload, and peak power (metabolic equivalents, Mets)] were recorded according to the Clinician’s Guide to Cardiopulmonary Exercise Testing statement [29]. O_2_-pulse, VO_2_ as a percentage of VO_2max_, and VO_2_ related to work rate (ΔVO_2_/ΔWR) were calculated (from beginning to VT_1_).

The VT_1_ is the first disproportionate increase in the rate of VCO_2_ compared with VO_2_; a significant increase in the V_E_/VO_2_ without a concomitant increase in V_E_/VCO_2_; or an increase in the PETO_2_ with no simultaneous decrease in the PETCO_2_ [30]. The VT_2_, also called the respiratory compensation point, is the first disproportionate increase in V_E_ compared with VCO_2_, the beginning increase in V_E_/VCO_2_, or the beginning decrease in the PETCO_2_ [30].

### Statistical analysis

The statistical analyses were carried out using SPSS 22.0. Baseline data are expressed as the mean and standard deviation. The differences between baseline data and post-intervention data are expressed as the mean and 95% confidence interval (CI). Comparisons of the baseline data and post-intervention changes among the four groups were performed using one-way analysis of variance (ANOVA) followed by Tukey’s post hoc test to identify significantly different means. ANOVA for repeated measurements of time course (pre- and post-intervention) and with or without NMN supplementation was also performed to defeat intergroup differences. In cases of a difference, the baseline measurement was used as the covariate in each covariance analysis. Cohen’s *d* was used to calculate effect sizes. An effect size of ≤0.2 was considered as indicating a small clinical effect, 0.5 as indicating a moderate clinical effect, and > 0.8 as indicating a large clinical effect [31]. Statistical significance was indicated by *p* <  0.05.

## Results

### The combination of exercise and NMN does not change body mass or alter body composition

All participants completed the required intervention. The baseline characteristics are shown in Table 1 and Supplementary Table S1. There were no significant differences among the four groups. Following the 6 weeks NMN supplementation in amateur runners, differences in body mass, BMI, or body fat% were found between the treatment groups and control group (Table 2).

**Table 1:** Participant baseline characteristics, including age, exercise years, anthropometric data, and VO_2max_

	total	control Group	Lower Dosage	Medium Dosage	High Dosage	P value
Age (years)	35.6 (6.1)	36.1 (6.0)	37.0 (5.7)	35.5 (6.1)	33.5 (6.6)	0.54
Exercise years (years)	2.8 (1.4)	2.7 (1.5)	2.6 (1.4)	2.9 (1.4)	2.8 (1.3)	0.94
Height (cm)	168.4 (6.1)	171.7 (6.3)	166.9 (5.1)	169.0 (6.2)	166.3 (6.3)	0.14
Body mass (kg)	62.5 (8.6)	64.9 (8.1)	62.0 (8.7)	62.5 (11.3)	60.7 (6.1)	0.69
BMI (kg/m2)	22.0 (2.6)	22.0 (2.6)	22.3 (3.2)	21.8 (2.9)	21.9 (1.6)	0.17
FFM (kg)	52.0 (7.5)	53.4 (7.2)	52.2 (7.1)	52.5 (8.4)	50.0 (7.5)	0.16
Body fat %	16.7 (6.7)	17.8 (5.7)	15.5 (6.4)	15.7 (6.6)	17.8 (8.4)	0.97
VO_2max_ ((L/min)	2.48 (0.49)	2.64 (0.46)	2.54 (0.52)	2.43 (0.45)	2.34 (0.52)	0.56

Note: Data in brackets represent means SD

**Table 2:** Changes in body composition of the participants after the 6-week intervention

	Control Group	Lower Dosage	Medium Dosage	High Dosage	Time P value	T × D P value
∆ body mass (kg)	0.15 (−0.42, 0.72)	0.07 (− 0.44, 0.57)	− 0.05 (− 0.78, 0.68)	0.63 (0.21, 1.04)	0.13	0.27
∆ BMI (kg/m2)	0.05 (−0.14, 0.25)	0.03 (− 0.15, 0.21)	− 0.02 (− 0.28, 0.24)	0.23 (0.08, 0.39)	0.11	0.24
∆ FFM (kg)	0.76 (0.17, 1.35)	−0.24 (− 0.95, 0.46)	−0.02 (− 0.72, 0.69)	0.31 (− 0.23, 0.85)	0.13	0.09
∆ body fat (%)	−0.94 (−2.11, 0.24)	0.47 (− 0.38, 1.31)	0.03 (− 1.04, 1.08)	0.16 (− 0.65, 0.96)	0.74	0.16

Note: ∆,The difference between pre and post intervention in mean (95% CI). ANOVA for repeated measurement for interaction of time (T) and dose (D)

### The combination of NMN and exercise increases VT but not VO_2max_ or O_2−_pulse

No significant changes in HR_**max**_, RER_**max**_, HRR, O_**2**_-pulse, peak power, peak workload, ∆O_**2**_/∆WR, or VO_**2max**_ were shown between the control and any of the NMN treatment groups. However, the VO_**2**_@VT_**1**_, %VO_**2max**_@VT_**1**_, HR@ VT_**1**_, power@VT_1_, and power@VT_2_ (Tables S1 and S2; Table 3) were increased significantly from the NMN supplementation compared with baseline, and the positive effect was in a dose-dependent manner (Table 4).

**Table 3:** Changes in cardiopulmonary function of the participants after the 6-week intervention from baseline

	control Group	Lower Dosage	Medium Dosage	High Dosage	Time *P* value	T × D P value
∆O2-pulse max (L/min/bpm)	0.73 (− 0.23, 1.68)	1.25 (0.23,2.27)	0.82 (0.07, 1.70)	1.17 (0.32, 2.02)	< 0.01	0.56
∆RER max	0.09 (0.00, 0.18)	−0.02 (− 0.07, 0.07)	0.05 (− 0.16, 0.11)	0.03 (− 0.08, 0.14)	0.04	0.36
∆ (L/min)	0.18 (0.02, 0.36)	0.23 (0.09,0.37)	0.26 (1.74, 0.35)	0.32 (0.20, 0.43)	< 0.01	0.48
∆ Peak power (Mets)	0.72 (−0.04, 1.49)	1.05 (0.45,1.65)	1.18 (0.78, 1.58)	1.45 (0.86, 2.06)	< 0.01	0.31
∆Peak workload (W)	10.9 (−2.47, 24.24)	11.25 (−0.43, 22.93)	13.58 (5.44, 21.7)	13.93 (8.92, 18.95)	< 0.01	0.58
∆HR@VT1 (bpm)	5.8& (0.3, , 11.4)	5.2& (1.1, 9.2)	12.8 (7.8, 17.8)	16.0 (10.4, 21.5)	< 0.01	< 0.01
∆O2-pulse @VT1 (L/min/bpm)	0.55 (−0.37, 1.46)	1.17 (0.71,1.62)	0.92 (0.17, 2.20)	2.00 (1.23, 2.77)	< 0.01	0.10
∆ @VT1 (L/min)	0.17#& (0.09, 0.24)	0.24& (0.18,0.30)	0.33 (0.25, 0.41)	0.47 (0.34, 0.60)	< 0.01	**< 0.01**
∆Power @AVT1 (Mets)	0.69#& (0.35, 1.03)	1.06& (0.80,1.31)	1.41& (1.02, 1.79)	2.13 (1.56, 2.69)	< 0.01	**< 0.01**
∆% @VT1 (%)	2.1& (−0.85, 1.49)	3.5& (0.05,7.00)	6.5 (3.62, 9.43)	10.3 (7.61,13.05)	< 0.01	**< 0.01**
∆HR@VT2 (bpm)	5.6 (1.9, 9.4)	6.7 (3.2, 10.2)	10.8 (4.5, 17.2)	12.4 (4.4, 20.5)	< 0.01	0.23
∆O2-pulse @VT2 (L/min/bpm)	0.55 (−0.37, 1.47)	1.58 (0.95,2.22)	0.50 (0.04, 1.91)	1.67 (0.72, 2.62)	< 0.01	0.16
∆ @VT2 (L/min)	0.20 (0.03, 1.49)	0.39 (0.26,0.52)	0.33 (0.22, 0.44)	0.44 (0.29, 0.58)	< 0.01	0.06
∆ Power @VT2 (Mets)	0.78 (0.07, 1.49)	1.78 (1.20,2.35)	1.56 (1.00, 2.11)	2.04 (1.34, 2.73)	< 0.01	**0.03**
∆% @VT2 (%)	1.55 (−1.75, 4.84)	6.67 (3.20,10.10)	4.25 (1.28, 7.22)	6.58 (3.57, 9.59)	< 0.01	0.06
∆O2/∆WR slope (ml/min/w)	0.11 (−0.58, 0.79)	0.44 (−0.16,1.04)	0.58 (0.01, 1.16)	0.69 (0.06, 1.45)	< 0.01	0.56

Note: The difference of parameter between pre and post intervention among four groups were performed using one-way ANOVA. #VS medium dosage, *P* <  0.05, &VS large dosage, *P* <  0.05

ANOVA for repeated measurement for interaction of time (T) and dose (D). bpm, beat per minute

**Table 4:** Analysis of the effect sizes (Cohen’s d), expressed as the mean (95% CI) and p value, for differences in adjusted means between the groups after the intervention

	Lower VS control		Medium VS control		High VS control		Medium VS Lower		High VS Lower		High VS Medium	
	ES (95%CI)	*P* value	ES (95%CI)	*P* value	ES (95%CI)	*P* value	ES (95%CI)	*P* value	ES (95%CI)	*P* value	ES (95%CI)	*P* value
VO2@VT1	0.61	0.16	1.45	<0.01	2.62	<0.01	0.83	0.05	2.03	<0.01	1.20	0.01
(L/min)	-0.25,1.42		0.48,2.30		1.43,3.62		-0.03,1.63		0.99,2.93		0.30,2.03	
%VO2max@VT1	0.34	0.42	0.89	0.04	1.56	0.01	0.55	0.19	1.22	0.01	0.69	0.10
(%)	-0.50,1.15		0.00,1.71		0.58,2.45		-0.28,1.34		0.31,2.04		-0.16,1.49	
HR@VT1	0.1	0.81	0.86	0.05	1.19	0.01	0.72	0.09	1.06	0.02	0.36	0.38
(bpm)	-0.72,0.92		-0.02,1.68		0.26,2.03		-0.13,1.52		0.17,1.87		-0.46,1.15	
Power @VT1	0.56	0.18	1.15	0.01	2.30	<0.01	0.58	0.16	1.74	<0.01	1.17	0.01
(Mets)	-0.29,1.38		0.23,1.99		1.18,3.25		-0.25,1.38		0.75,2.61		0.27,1.99	
Power @VT2	0.99	0.02	0.77	0.07	1.25	0.01	0.22	0.59	0.26	0.52	0.48	0.24
(Mets)	0.09,1.82		-0.10,1.59		0.32,2.09		-0.59,1.01		-0.55,1.05		-0.35,1.27	

*bpm* beat per minute

### Adverse events

During the intervention period, all participants had taken the NMN or placebo according to the requirements, and none of the participants reported an adverse event. No obvious abnormalities were shown on the ECG during exercise in the CPET.

## Discussion

In rodents, NMN is able to increase cellular NAD^+^ content in dosage ranges from 31.25 mg/kg/d to 500 mg/kg/d, and it is shown to be safe [2, 6, 22]. In humans, only one study of this has been reported, which used a single dose of 500 mg NMN. In this study, the NMN increased circulatory NAD^+^ and was shown to be safe [32]. Here, we administrated three dosages (300, 600, and 1200 mg/d) of NMN supplementation to healthy amateur runners during a 6-week exercise training program. The main finding of this study is that NMN supplementation during exercise improved first ventilatory threshold (VT_1_) and power@VT_2_ without changing the VO_2max_ and that this improvement was dose-dependent.

### Exercise combined with NMN did not change body composition

Our observations indicate that six-week aerobic exercise with low- to high-dose NMN supplementation did not alter body mass, FFM, BMI, or body fat%. Both NR [33] and NMN [7, 22] inhibited high-fat diet or age-induced weight gain and increased energy consumption in mice. A human study [34] of NR on body composition in healthy obese middle and older people showed that body fat % improved, whereas body weight remained unchanged, and the improvement appeared to be a gender-dependent. Exercise combined with NR showed no additional improvement in body weight reduction and fat deposits in young mice compared to exercise only [27]. Considering the good standing of BMI and body fat % of the participants in this study, no further changes in BMI and body fat % were expected.

### Exercise combined with NMN increases VT but not VO_2max_

A recent study on rodents demonstrated that exercise combined with NMN led to a further increase in running endurance in healthy young mice [17]. Our results specifically reveal that 6 weeks of endurance exercise combined with NMN supplementation in amateur runners enhanced VT_1,_ VO_2_, and VO_2max%_ but not VO_2max_, V_Emax_, O_2_-pulse, ∆O_2_/∆WR, RER, or peak power. In addition, a large dose of NMN also improved the power@VT_2._ These results indicate that NMN supplementation was able to further increase the ventilatory threshold compared to exercise alone. The improvement may be attributed to an improved ability of O_2_ utilization by skeletal muscle, as no changes in VO_2max_ and O_2−_pulse and ∆O_2_/∆WR were observed instead of improvement of cardiac function. Our data suggest that skeletal muscle is one of the most sensitive tissues to NMN in humans.

It is well-known that endurance exercise increases aerobic capacity through improving mitochondrial function, vascular endothelium function, and capillary density of muscle, independent of age [35]. NMN administration had also been reported to improve mitochondrial function in various metabolic organs, including skeletal muscle [36, 37], to improve vascular endothelium function [17, 38], to promote neoangiogenesis and increase capillary density, blood flow, and soluble oxygen levels, and to switch skeletal muscle fibers to the more oxidative type in elderly or aged mice [17, 18]. These exercise-induced adaptive changes undoubtedly contribute by increasing the anaerobic threshold [39]. Exercise combined with NMN administration further increased exercise endurance and rebuilt the skeletal muscle capillary number and density to youthful levels in elderly mice, and it significantly increased the capillary/myofiber ratio in the quadriceps compared to NMN alone or exercise alone in young mice [17].

Although NMN supplementation improved endothelium function and reduced vessel wall stiffness in aged rodents [38] and restored cardiac function in cardiac pathologies, such as cardiomyopathy and ischemia/reperfusion (I/R) cardiac injury [40, 41], research shows that NMN supplementation does not affect cardiac function or cardiac capillary density in elderly mice [17]. Our results also indicate that exercise with NMN supplementation had no added effect on cardiac function in amateur runners.

### Dose-dependent relationship

It was reported in mice that lower dosages of NMN (100 mg/kg/d) were better compared with larger doses (300 mg/kg/d) for body weight, body composition, insulin sensitivity, bone mineral density, and physical activity [7], and treatment with 62.5 mg/kg NMN was better than that with 125–500 mg/kg NMN for ischemia-induced brain damage [8]. Furthermore, a lower dose of NMN led to an improvement in female infertility with enhanced oocyte quality [42]. According to an equivalent surface area dose (mg/kg body weight) conversion, a human dose is approximately one-tenth of that for a mouse [7]. Therefore, 50, 100, and 200 mg/kg/d of NMN for mice is approximately 300, 600, 1200 mg/d for humans of 60 kg body weight. In contrast, our human study showed that exercise combining with NMN supplementation increases VT in a dose-dependent and the large dose of NMN had better effect. Of note, we also measured physical function and the results show that exercise combined with NMN supplementation had no effect on grip strength, push-up, or sit-and-reach compared with exercise only, but 600 mg/d NMN, not 1200 mg/d NMN, significantly improved single leg stance test results (Supplementary Tables S3–S5). In one study, it was reported that there was an overdose NAD^+^ precursor reduced skeletal muscle NAMPT content through negative feedback [43]. All of these data suggest that for NMN, there may not exist as one-size-fits-all.

### Side effects

Pharmacological dose of NA and nicotinamide (NAM) may result in painful flushing, liver damage and NAM exhibit sirtuin-inhibiting effects [44, 45], whereas NMN and NR exhibit better pharmacokinetic and pharmacological properties [17, 46–48]. In rodent, long-term NMN administration from 100 to 300 mg/kg/d did not result in any obvious side effects [7]. In human, single oral dose ranging from 100 to 500 mg NMN was safe and had no significant deleterious effects in healthy individual [32]. In this study, 300–1200 mg, a day, 6 week did not have any obvious adverse symptoms and abnormal ECG.

### Limitations

In this study, one limitation was conducted the CPET on a cycloergometer instead of a treadmill. Taking into account the modality including cycling and the advantages of a cycloergometer recording the ECG, we had chosen a cycloergometer. However, the running was the main form of modality, CPET on a treadmill ergometer would be a better way to assess aerobic fitness. In addition, it was reported that values of VO_2max_ and the VT in active runners or amateur triathletes were ~ 10% higher on treadmill than cycle ergometry [49], thus we prescribed targed HR during running training corresponding to 100–120% VO_2_ at VT_1_ measured in cycle ergometer. Nevertheless, the intensity prescription for running training in the context of our study subjects did not rule out bias because of individual differences of interchangeability between tests.

The second limitation was that the changes of blood lactate (especially blood lactate at the peakpower) was not simultaneously measured during CPET. NMN supplementation had been reported to reduce post-exercise blood lactate with improvement in endurance in mice [17]. Another research study showed that three-week swimming training combined NR administration (300 mg/kg/d) reduced glucose concentration and maximal blood lactate accumulation in rats with a decrease in exercise endurance [27]. The measurement of blood lactate could provide another insight into the metabolic adaptations underlying the VT-improving effect.

Another limitation of the current investigation was separate female from male due to a limited number of female participants. Additional studies are needed to determine if there exist gender difference and improvement in vascular endothelium function, and whether the combination of NMN supplementation and exercise leads to increases in capillary density, blood flow, and mitochondrial function.

## Conclusion

The results of this study reveal that exercise training combining with the supplementation of NMN further lift ventilatory threshold in amateur runners, the benefit is dose-dependent and muscle-related.

### What are the new findings

- ▸The combination of NMN supplementation and exercise further improves ventilatory threshold even among healthy young and middle-aged people.
- ▸The improvement of aerobic capacity is in a dosage-dependent, large dosage of NMN with exercise has better effects.
- ▸The improvement is muscle, not cardiac, related.

### How might impact on clinical practice in the near future

- ▸NMN as adjunct treatment may help to improve performance during exercise training.
- ▸Exercise training combining with NMN supplementation may be a novel and practical strategy to increase endurance performance of athletes.

## Supplementary Information

**Additional file 1: Table S1.** Baseline cardiopulmonary function parameters of the participants. **Table S2.** Changes in cardiopulmonary function after 6-week intervention from baseline. **Table S3.** Baseline results of the physical function test. **Table S4.** The change in the physical function test results at 6-week intervention from baseline. **Table S5.** Analysis of the effect sizes (Cohen’s d), expressed as the mean (95% CI) and *p* value, for differences in adjusted means between the groups after the intervention.
