# Supplementary material for: Nicotinamide mononucleotide supplementation enhances aerobic capacity in amateur runners: a randomized, double-blind study
Source: J Int Soc Sports Nutr. 2021 Jul 8;18:54. doi: 10.1186/s12970-021-00442-4 (PMC8265078; doi:10.1186/s12970-021-00442-4)
Supplement: Supplementary file 1 — Additional file 1: Table S1. Baseline cardiopulmonary function parameters of the participants. Table S2. Changes in cardiopulmonary function after 6-week intervention from baseline. Table S3. Baseline results of the physical function test. Table S4. The change in the physical function test results at 6-week intervention from baseline. Table S5. Analysis of the effect sizes (Cohen’s d), expressed as the mean (95% CI) and p value, for differences in adjusted means between the groups after the intervention. [file 12970_2021_442_MOESM1_ESM.doc]

**Supplementary materials**

Table S1. Baseline cardiopulmonary function parameters of the participants

|  | control Group | Lower  Dosage | Medium  Dosage | High  Dosage | P  value |
| --- | --- | --- | --- | --- | --- |
| Rest HR(bpm) | 67(10) | 67(6) | 67(10) | 68(7) | 0.96 |
| SBP(mmHg) | 115.6(9.5) | 115.8(7.6) | 114.3(7.1) | 110.3(7.4) | 0.30 |
| DBP(mmHg) | 66.6(10.6) | 71.6(7.8) | 69.6(5.7) | 71.8(8.1) | 0.40 |
| HR max(bpm) | 163(15) | 169(11) | 161(16) | 163(14) | 0.59 |
| HRR(bpm) | 97(8) | 101(13) | 94(14) | 96(12) | 0.45 |
| VEmax(L/min) | 81.6(17.9) | 76.3(16.7) | 73.7(19.2) | 73.5(16.6) | 0.67 |
| O_2_-pulse @VT_1_ (L/min/bpm) | 13.0(2.6) | 11.9(2.7) | 12.53.0) | 11.2(2.3) | 0.39 |
| O_2_-pulse @VT_2_ (L/min/bpm) | 15.1(3.2) | 13.8(2.8) | 14.6(3.3) | 13.3(2.8) | 0.48 |
| O2-pulse max (L/min/bpm) | 16.1(2.9) | 15.1(3.0) | 15.7(2.9) | 14.3(2.6) | 0.49 |
| RER max | 1.27(0.14) | 1.36(0.09) | 1.34(0.10) | 1.33(0.14) | 0.37 |
| Peak power (Mets) | 11.8(1.7) | 11.5(2.0) | 11.4(2.0) | 11.1(2.1) | 0.79 |
| Peak workload (W) | 223.4(43.7) | 218.5(37.2) | 206.2(40.9) | 197.9(45.2) | 0.45 |
| HR@VT1 (bpm) | 129(15) | 136(13) | 126(14) | 123(11) | 0.10 |
| @VT1(L/min) | 1.65(0.35) | 1.61(0.33) | 1.50(0.34) | 1.38(0.30) | 0.22 |
| Power @VT1(Mets) | 7.4(1.1) | 7.6(1.2) | 7.2(1.8) | 6.6(1.2) | 0.35 |
| %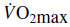 @VT1 | 63.0(5.5) | 63.7(4.0) | 61.9(6.9) | 59.1(1.2) | 0.15 |
| HR@VT2 (bpm) | 152(15) | 159(12) | 148(17) | 150(13) | 0.33 |
| @VT2(L/min) | 2.29(0.43) | 2.16(0.42) | 2.06(0.49) | 1.99(0.49) | 0.44 |
| Power @VT2(Mets) | 10.3(1.6) | 10.1(1.5) | 9.7(2.1) | 9.5(1.8) | 0.64 |
| %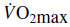 @VT2 | 87.6(4.4) | 85.5(5.9) | 84..9(5.9) | 84.8(5.2) | 0.52 |
| ∆ O2/∆WR slope (ml/min/w) | 10.8(0.7) | 10.6(1.0) | 10.8(0.6) | 10.6(0.8) | 0.89 |

Note: bpm, beat per minute; Data in brackets indicate SD

Table S2. Changes in cardiopulmonary function after 6-week intervention from baseline

|  | control  Group | Lower  Dosage | Medium  Dosage | High  Dosage | Time  P value | T×D  P value |
| --- | --- | --- | --- | --- | --- | --- |
| ∆HR max  (bpm) | 4.7  （1.1，8.4） | 4.8  （1.3，6.2） | 7.4  （1.7，13.1） | 8.3  （1.8，14.8） | <0.01 | 0.25 |
| ∆HRR  (bpm) | 5.0  （1.7，8.5） | 2.3  （-2.6，7.1） | 6.5  （0.2，12.8） | 7.8  （0.5，15.1） | <0.01 | 0.47 |
| ∆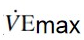  (L/min) | 10.65  （2.59，18.71） | 18.50  （8.92,28.07） | 11.67  （3.82，19.52） | 18.99  （10.41，27.56） | <0.01 | 0.29 |

Note : The difference of parameter between pre and post intervention among four groups were performed using one-way ANOVA. #VS medium dosage,P<0.05, &VS large dosage,P<0.05.

ANOVA for repeated measurement for interaction of time (T) and dose (D).

Physical function

To begin testing, the participants performed a generalized warm up consisting of callisthenic and dynamic stretching exercises, lasting 5 - 10 minutes. After the warm up, the participants commenced the grip strength, push-up, sit-and-reach test and single leg stance test.

Grip strength test: the grip strength was measured using a digital handgrip dynamometer. The participants held the dynamometer in the dominant hand to be tested, then squeezed the dynamometer with maximum isometric effort for at least 5 seconds. No other body movement was allowed. The better of two attempts was recorded.

Push-up test: the males performed the standard push-up using the toes as the pivotal point. The females did a modified push-up using the knees as the pivotal point. The participants bent the elbows until the elbows bent to 90 degrees, then returned to the starting position with the arms extended. As many complete push-ups as possible were performed. The number of completed push-ups was recorded.

Sit-and-reach test: the participants were required to sit on the floor with legs fully extended and with feet flat against the test box, then slowly bent forward and reached along the top of the ruler as far as possible. The better of two attempts was recorded.

Single leg stance test: the participants were instructed to stand on one leg unassisted with their eyes closed. The time when one foot is flexed to the time when it touches the ground or moves out of position was recorded. The test was repeated twice for the same leg. Then, the foot of the other leg was repeated the same. The best performance was recorded.

The results for all test are shown in table S3, table S4, and table S5.

Table S3. Baseline results of the physical function test

|  | control Group | Lower Dosage | Medium Dosage | High Dosage | P value |
| --- | --- | --- | --- | --- | --- |
| Single leg stance test(S) | 32.8（35.1） | 73.7(57.7) | 33.2(27.0) | 50.4(40.2) | 0.07 |
| Grip strength (Kg) | 38.9 (7.7) | 38.5(7.3) | 40.3(10.8) | 41.8(8.1) | 0.78 |
| Push-ups | 23.4(10.5) | 30.8(9.4) | 36.1(18.6) | 34.2(13.1) | 0.12 |
| Sit-and-reach (cm) | 6.1(10.7) | 6.1(7.2) | 8.3(7.2) | 8.8(8.1) | 0.79 |

Note: Data in brackets means SD

Table S4. The change in the physical function test results at 6-week intervention from baseline

|  | control  Group | Lower  Dosage | Medium  Dosage | High  Dosage | Time  P value | T×D  P value |
| --- | --- | --- | --- | --- | --- | --- |
| ∆ Single leg stance test(S) | 16.9  （2.9，30.9） | 3.1  （-22.0，28.0） | 37.7  （9.7，65.7） | -1.7  （-23.5，20.1） | 0.01 | 0.04 |
| ∆Grip strength (Kg) | 1.1  （-0.5，2.6） | 0.5  （-1.3，2.2） | 0.9  （-2.2，3.9） | 0.8  （-1.0，2.5） | 0.07 | 0.98 |
| ∆Push-ups | 5.2  （2.9，7.4） | 1.2  （-2.0，4.4） | 0.1  （-4.3，4.5） | 3.8  （-2.2，9.7） | 0.85 | 0.23 |
| ∆Sit-and-reach (cm) | 1.1  （-0.8，3.1） | 2.2  （0.8，3.6） | 0.9  （-0.8，2.6） | -0.9  （-2.6，0.7） | 0.74 | 0.05 |

Note: ∆ ,The difference between pre and post intervention in mean(95% CI). ANOVA was used to repeated measure the interaction of time (T) and dose (D).

Table S5. Analysis of the effect sizes (Cohen’s d), expressed as the mean (95% CI) and p value, for differences in adjusted means between the groups after the intervention

| Lower VS Control Medium VS Control High VS Control Medium VS Lower High V S Lower High V Medium | | | | | | | | | | | | |
| --- | --- | --- | --- | --- | --- | --- | --- | --- | --- | --- | --- | --- |
|  | ES P  (95%CI) value | | ES P  (95%CI) value | | ES P  (95%CI) value | | ES P  (95%CI) value | | ES P  (95%CI) value | | ES P  (95%CI) value | |
| Single leg stance test(S) | -0.11  -0.91,0.69 | 0.79 | 0.59  -0.24,1.39 | 0.15 | -0.41  -1.20,0.42 | 0.32 | 0.69  -0.15,1.49 | 0.11 | -0.29  -1.08,0.53 | 0.47 | -1.0  -1.82;-0.12 | 0.02 |
